# The ‘Dark Side’ and ‘Bright Side’ of Personality: When Too Much Conscientiousness and Too Little Anxiety Are Detrimental with Respect to the Acquisition of Medical Knowledge and Skill

**DOI:** 10.1371/journal.pone.0088606

**Published:** 2014-02-27

**Authors:** Eamonn Ferguson, Heather Semper, Janet Yates, J. Edward Fitzgerald, Anya Skatova, David James

**Affiliations:** 1 School of Psychology, University of Nottingham, Nottingham, United Kingdom; 2 Centre for Health Psychology, Staffordshire University, Stoke on Trent, United Kingdom; 3 Medical Education Centre, University of Nottingham School of Medicine, Queen's Medical Centre, Nottingham, United Kingdom; 4 Department of General Surgery, Barnet Hospital, Barnet, United Kingdom; 5 Horizon Digital Economy Research, Nottingham University Innovation Park, Nottingham, United Kingdom; Tokai University, Japan

## Abstract

Theory suggests that personality traits evolved to have costs and benefits, with the effectiveness of a trait dependent on how these costs and benefits relate to the present circumstances. This suggests that traits that are generally viewed as positive can have a ‘dark side’ and those generally viewed as negative can have a ‘bright side’ depending on changes in context. We test this in a sample of 220 UK medical students with respect to associations between the Big 5 personality traits and learning outcomes across the 5 years of a medical degree. The medical degree offers a changing learning context from pre-clinical years (where a more methodical approach to learning is needed) to the clinical years (where more flexible learning is needed, in a more stressful context). We argue that while trait conscientiousness should enhance pre-clinical learning, it has a ‘dark side’ reducing the acquisition of knowledge in the clinical years. We also suggest that anxiety has a ‘bright side’ enhancing the acquisition of skills in the clinical years. We also explore if intelligence enhances learning across the medical degree. Using confirmatory factor analysis and structural equation modelling we show that medical skills and knowledge assessed in the pre-clinical and clinical years are psychometrically distinguishable, forming a learning ‘backbone’, whereby subsequent learning outcomes are predicted by previous ones. Consistent with our predictions conscientiousness enhanced preclinical knowledge acquisition but reduced the acquisition of clinical knowledge and anxiety enhanced the acquisition of clinical skills. We also identified a curvilinear U shaped association between Surgency (extraversion) and pre-clinical knowledge acquisition. Intelligence predicted initial clinical knowledge, and had a positive total indirect effect on clinical knowledge and clinical skill acquisition. For medical selection, this suggests that selecting students high on conscientiousness may be problematic, as it may be excluding those with some degree of moderate anxiety.

## Introduction

There is a growing awareness among psychologists, economists and biologists that personality traits play an important role with respect to predicting major life outcomes (e.g., educational attainment), economic markers (e.g., GDP) and social capital [1]–[5]. However, conclusions in this literature often take the form of validity generalization statements and examine associations at a general domain level of the trait (although work examining traits at the facet level is emerging), concluding that certain traits, like conscientiousness (C) are beneficial and others, like anxiety, are not [2]–[3], [6]–[7]. However, there is a growing realisation that at a domain level, traits like conscientiousness also have a ‘dark side’ [8] and traits like anxiety and other negative traits, like narcissism, have a ‘bright side’ [9]–[10]. This is consistent with the theoretical position that personality traits evolved with high scores manifesting both costs and benefits and that a trait's effectiveness depends on how these costs/benefits match with the on-going context [11]. The same is not true for intelligence, where higher intelligence should be beneficial for an individual across all contexts [12].The first aim of this paper is to test these conjectures by examining the influence of personality and intelligence on learning outcomes across the changing context of medical training. While medical training concerns the acquisition of medical expertise, in terms of the successful application of knowledge and skills, research is only just starting to examine if medical skills and knowledge are psychometrically distinct constructs [13]. Thus the second aim of this paper is to extend this literature on knowledge and skill acquisition in medical training [14] by examining if clinical knowledge and clinical skills are psychometrically separable and if so how these are influenced by personality, intelligence, knowledge and demographics [15].

### The ‘learning backbone’ and medical knowledge and skill

The study reported is set within a 5 year medical degree at Nottingham University, UK [16]. Across these 5 years both (1) medical knowledge (i.e., acquisition of factual information) and (2) skills (i.e., practical skills to examine, make a diagnosis, and interact with a patient) are developed and assessed. In the first two pre-clinical years of medical training students mainly learn the ‘basic sciences’ via standard didactic learning, with assessment by exams. In the 3^rd^ pre-clinical year they undertake a piece of empirical work (assessed by thesis and an oral examination) plus taught courses, followed by a 6-month introductory clinical course (Bachelor of Medical Science: B Med Sci). In their final two years, training in clinical practice is undertaken [16]–[17]. In these last two clinical years students have considerable patient contact and have to apply the knowledge and skills learnt over the first three pre-clinical years to diagnosis and treatment. Assessment in these final years is via a series of exams designed to assess ‘clinical knowledge’ as well as a series of direct observations of clinical skills via Objective Structured Clinical Examinations (OSCEs) and Objective Structured Long Examination Records (OSLERs), designed to assess ‘clinical skills’. Thus, depending on the year of study it is possible to distinguish between pre-clinical knowledge assessed over years 1 to 3 (marks form years 1 and 2 and the B Med Sci mark), clinical knowledge assessed in years 4 and 5 and clinical skills assessed also in years 4 and 5 by OSLERs and OSCEs.

The assessment of pre-clinical and clinical knowledge represents factual knowledge and the OSCEs and OSLERS assessment of skill. These map nicely onto distinctions between knowledge (declarative knowledge or *what* is known) and skill (procedural knowledge, or knowing *how* to do something) in models of knowledge and skill acquisition [14], [18]–[19]. With respect to skill acquisition these models [14] suggest that at the most basic level procedural knowledge represents the reproduction of simple learned behaviours. This then progresses via training to a ‘compilation’ stage where this initial skill develops to become faster and exhibit fewer errors through practice and finally progressing to a stage of ‘tuning’ and automaticity [14], [19]. Medical students in the final two years are likely to be in the compilation stage and as such direct observation via OSCEs and OSLERs is an appropriate assessment tool [19]. As clinical knowledge and clinical skills represent different aspects of medical training, they should be related yet distinct components of training and learning [19].

This distinction between preclinical knowledge, clinical knowledge and clinical skills should be distinguishable psychometrically, with these aspects of learning forming separate factors in a factor analysis. To date, the majority of work in the area of medical training has not attempted psychometrically to distinguish performance in assessments of pre-clinical knowledge, clinical knowledge and clinical skills. One recent exploratory factor analysis identified factors that were not based on knowledge and skill, but rather identified two factors representing when the assessments were conducted [13]. The first factor represented assessments conducted in the pre-clinical years and the second factor assessments conducted in the clinical years [13]. Nonetheless, such exploratory factor models do not allow hypothesis testing. Therefore, in this study we use confirmatory factor analytic models, which allow for hypothesis testing, to compare a series of factor models to see which fits these data best. We specified four models: (1) a single factor model (all assessments of knowledge and skill load on a single factor) termed the ‘single factor model’, (2) a two factor model that differentiates whether assessments conducted in the preclinical years load onto a different factor to those conducted in the clinical years [13], termed the ‘two factor temporal model’, (3) a two factor model that distinguishes knowledge (pre-clinical and clinical knowledge assessments form one factor) and clinical skill (OSCE and OSLER scores form the other factor) termed the ‘two factor knowledge and skills’ model and (4) a three factor model that differentiate pre-clinical knowledge, clinical knowledge and clinical skill, termed the ‘three factor model’.

Establishing this distinction is important as research is now starting to examine differential predictors (demographics and traits) of clinical knowledge and clinical skill [15] and as such showing that they are psychometrically distinct is crucial. Furthermore, McManus et al [13] define the ‘learning backbone’ for medicine as the extent to which current and subsequent learning is dependent of learning at an earlier stage. This ‘learning backbone’ progresses from the first exams of general academic knowledge that the students take in the UK at 16 years of age (The General Certificate of Secondary Education: GCSE), to their Advanced level (A level) exams at 18 years of age prior to entry to medical school, through to learning in medical school that progresses from pre-clinical knowledge to clinical knowledge and clinical skills. Statistically this backbone represents a pattern of correlations over time which has similarities to a simplex [13], [20]–[22], in that subsequent learning is predicted by all previous learning. Simplexes can occur when the same variable is assessed over time (learning) with the association between adjacent learning outcomes being stronger than more distal ones. The causes of a simplex are open to debate [21]–[22]. Here while ordered over time the content of the learning also changes (general knowledge, pre-clinical knowledge, clinical knowledge, clinical skill etc.), thus it may be more appropriate to refer to this as simplex-like, as the content of what is assessed, as well as the order in which it is assessed , will influence the pattern of associations. Thus given that pre-clinical knowledge, clinical knowledge and clinical skills are part of this learning backbone, it is helpful to show that they are distinct aspects of learning.

### Personality, intelligence, costs-benefits, context and learning

The medical degree context changes from a relatively safe classroom based education in the pre-clinical years (1 to 3), to one where decisions matter and the context becomes more threatening, accompanied by a change in teaching and the learning environment in the clinical years (4 to 5) [23]. Indeed, there is evidence that medical students find the transition from the pre-clinical to clinical years stressful [24] and learning styles change with a greater reliance on strategic and deep learning and less on surface learning in the clinical years [25]–[26]. We are not suggesting that the pre-clinical learning context involves simply rote learning of facts, but rather that compared to the clinical learning context learning of facts and methodical approach may pay relatively more dividends pre-clinically.

This changing context provides an opportunity to examine if a trait that affords a benefit at one stage of the degree (e.g., enhancing learning) will also entail a cost (e.g., reduced learning) at a different stage of the degree. Table 1 provides a description of the costs and benefits of scoring at the high end on each of the Big 5 personality traits [27] and is taken from and draws together the work by Nettle [11] and Widiger and Mullins-Sweatt [28].

**Table 1:** Predictions for Personality and a Function of Changing Context.

	Nettle (2007) [11]		Widiger and Mullins-Sweatt (2009) [28]	
	Costs	Benefits	Abnormally High	Normally High
S	Exposure to physical risks	Social allies and environmental exploration	Foolhardy, reckless, manic	Affectionate, energetic, adventurous
Low ES	Negative response to stress, susceptibility to depression	Vigilance to danger, competitiveness	Fearful, depressed	Vigilant, wary, vulnerable
I	Psychosis proneness, unusual beliefs	Creativity (enhancing attractiveness)	Bizarre interests, lives in fantasy, eccentric	Imaginative, creative, curious
A	Subject to cheating	Attention to others mental states	Gullible, docile, meek	Empathic, trusting, cooperative
C	Obsessionality, rigidity	Attention to long term fitness benefits	Perfectionist, single-minded doggedness	Efficient, organized, purposeful. ambitious

*Note*. S = Surgency, ES = Emotional Stability, I = Intellect, A = Agreeableness and C = Conscientiousness. The schema for costs and benefits is derived from Table 1 in Nettle (2007) [11] and Figure 1 in Widiger and Mullins-Sweatt [29]. McCrae and Costa [60] also present characteristic of low and high scorers on each Big 5 trait, but the above scheme subsumes their characteristics of each trait at the high end of that trait.

Nettle [11] identifies the costs and benefits associated with each of the Big 5 traits at the domain level and Widiger and Mullins-Sweatt [28] distinguish maladaptively high and normal high levels of each trait (as well as normal and maladaptively low levels) at their facet level and the behaviours associated with these. Comparing these two frameworks suggests that benefits are associated with normal high levels of a trait and costs with maladaptively high levels. One implication of this is the need to be aware of the range of scores recorded on the personality measure to help aid interpretation of any findings with respect to costs and benefits. The main problem, however, is at present there are no clear guidelines to distinguish when a level of a trait should be considered maladaptively or normally high [28]–[30]. In the absence of any guidelines we provide a purely descriptive aid to interpretation by comparing the range of each of the Big 5 traits in our sample of medical students with a group of non-medical students from the same University.

A second implication of the Widiger and Mullins-Sweatt [28] framework (see Table 1) is that curvilinear associations may be expected between each of the Big 5 traits and an outcome. For example, vigilance as a characteristic of normal high levels of anxiety, with maladaptively high levels of anxiety (neuroticism) associated with greater fear. This suggests that extreme high (very calm and relaxed) and low (fearful) levels of Emotional Stability (ES) may be linked to poor performance, but moderate-high levels of ES may be associated with good performance (the bright side). For Conscientiousness (C), maladaptively high levels, with single-minded doggedness, should be related to reduced performance, especially when flexibility is needed. Similarly lower levels of C (e.g., dis-organized, lax, careless) should be related to reduced performance, whereas a normal high level associated with being organized and methodical may be beneficial. These curvilinear possibilities are purely exploratory in nature, especially as it is not clear exactly what score marks the transition of each trait from maladapatively high (low) to normal high (low).

With respect to the changing context across medical training the methodical and ordered thought and perfectionism associated with normal high levels of C should be beneficial in the pre-clinical years, where it should aid success when surface learning is likely to be beneficial. Indeed, C is more strongly linked to reproduction-directed learning (e.g., rehearsing), than flexible learning styles [31] but is also associated with a potential for strategic learning [32]. However, in the clinical years, greater flexibility and adaptation is required. The rigidity of thought associated with high C may be a hindrance in this context. One study has previously reported this pattern [20], with others suggesting it needs replicating and extending [33]. With this in mind this study extends Ferguson et al. [20] by examining if this pattern for C remains in the presence of the other Big 5 traits, intelligence, socio-demographic controls and extensive general academic knowledge acquired prior entering medical school, which were not controlled in Ferguson et al. [20]. The study by Ferguson et al [20] is also extended to examining how traits affect pre-clinical knowledge and, clinical knowledge as well as clinical skill and to explore the possibility of curvilinear effects.

The other four traits (not withstanding potential curvilinear effects) should enhance performance in the clinical years specifically. Those high in surgency (S), who benefit from more social allies and environmental exploration and adventurousness, should benefit more in the clinical years, when it is necessary to work with others and take risks by exploring a new and changing context. Similarly, the creativity and intellectual engagement [34] associated with normal high levels of intellect (I) should be helpful in the clinical years, when creative problem solving is required. While they are related, the trait intellect should be distinguished from intelligence [34], as the former reflects a person's typical behaviour, disposition and preference towards creative pursuits, curiosity and imagination, whereas intelligence represents maximal performance in terms of reasoning and problem solving across domains (e.g., spatial, numerical, verbal). The benefits of being collegiate in medicine often outweigh the cost of going alone, especially in high pressured clinical settings. Furthermore, good perspective taking skills should also enhance bedside manner and medical history tasking during OSCEs and OSLERs. As those high in agreeableness (A) are more likely to be cooperative [35] and have good perspective taking skill [36], higher levels of A should be associated with enhanced clinical skills performance. Increased vigilance has been identified as a key to clinical success [37]. Those low in ES (or high anxiety/neuroticism), especially at moderate low levels, should show increased vigilance and should be observed to be more successful in their clinical years (a ‘bright side’ of anxiety).

Finally, the separate assessment of clinical knowledge and skills provides the opportunity to examine their associations with personality [15]. Specifically performing a medical skill (e.g., taking blood) has implications for patient and physician safety, in a way that acquiring knowledge does not. As such, medical skill performance is likely to be more anxiety provoking and require more vigilance. The concept of ‘defensive direction’ and increased vigilance associated with moderate-low ES suggests that moderate-low ES should be linked specifically to clinical skill acquisition [9]. With respect to C, evidence suggests that it is more likely to be associated with knowledge acquisition [38] and it is predicted that this association will be negative for clinical knowledge. A recent paper, however, showed that C is positively associated with both clinical knowledge and clinical skill [15]. However, that study looked at C as a predictor of clinical skill and clinical knowledge separately and did not control for the degree of correlation between the two or the learning backbone. The study reported here does both.

### Influence of General Knowledge and Demographics

Students are selected into medicine in the UK based on their levels of previous academic knowledge (GCSEs and A levels), as assessed by National school exams [17]. These examinations test knowledge across a variety of subjects at 16 years of age (GCSE) and more specific knowledge at 18 years of age (A level exams) and these are seen to reflect general academic knowledge. Others have shown that general academic knowledge (A levels) is a more important predictor of medical school attainment than intelligence [39]. However, that study examined overall attainment, but did not differentiate pre-clinical knowledge and clinical knowledge and clinical skills (see also [13]). Also effects of personality were not controlled. Thus, the analyses reported here examine the effect of prior general knowledge on attainment in medical training in a more detailed manner.

Similarly there are well known demographic predictors of medical school performance, with women performing better than men [40]–[42] as do white students compared to non-white students [42]. These factors are also examined in the analyses reported.

### The Current Paper

The aim of the current paper is to test a prediction from the cost-benefit model of personality [11] with respect to C, by exploring if C enhances pre-clinical knowledge and inhibits clinical knowledge (has a ‘dark side), in the presence of the other 4 Big 5 traits, intelligence and general knowledge. We also explore if there is a ‘bright side’ to anxiety by exploring if moderate-low ES predicts improved clinical skills. We compare this to intelligence, which should have a positive effect across the whole medical degree. The current paper also adds to the literature by examining a variety of factor models concerning the distinction between pre-clinical knowledge, clinical knowledge and clinical skill.

## Methods

### Samples

#### Main Analysis

The main 5 year longitudinal sample for this paper initially consisted of 243 UK medical students starting their 5 year degree in 2003, graduating in 2008. Years 1 and 2 represent pre-clinical training assessed by exams, in year 3 they complete their B Med Sci and in years 4–5 they complete OSCE and OSLER assessments of clinical skills and exam based measures of clinical knowledge. Of these, 220 medical students were entered into the final analyses, with a mean age of 18.5 years (SD = 1.3) of which 63% were female and 75% were white. Participants were removed due to missing data on all assessments (due to death or dropped out), not completing their clinical years or having 50% of their final year marks missing. The main path of interest to replicate from Ferguson et al [20] is from C to clinical knowledge and was −.20. Treating this as a partial beta and that in the predicted model there would potentially be 7 independent predictors, a minimum N of 102 subjects are needed [43]. As such, the sample size is sufficient for the analyses reported here. There were no missing data on the academic assessments, the Big 5 and intelligence. There was a small amount of missing data on ethnicity (N = 6), A levels (N = 5) and GCSEs (N = 19). Full information maximum likelihood (FIML) procedures were used to impute missing data. Participants voluntarily completed measures of the Big 5 and intelligence (as part of a larger psychometric battery) within the first two weeks of starting their medical degree. Importantly participants had not been selected on the basis of the scores on the Big 5 or intelligence.

#### Comparison Sample

To aid interpretation of the Big 5 in the main sample of medical students we compared the means, standard deviations and range of scores observed for the medical students with other students at the same University not studying medicine. While this provides a purely descriptive comparison only, it at least allows us to see if medical students, in this sample, have any variation in range of scores compared to non-medical students. The comparison sample consists of an opportunity sample of 465 Nottingham University students (27 were medical students) with a mean age of 20.3 (SD = 2.3) years (43% male) and were sampled in 2008. This sample was part of a larger study examining personality, motivation and degree choice and all undergraduates who took part were entered a prize draw of £75 (equivalent to $115) (see [44] for a fuller description of the sampling procedures and other measure completed.). All 465 completed the same measure of the Big 5 (Goldberg's, 1992 bi-polar markers [27]) as the main medical student sample. Of these 219 completed a paper and pencil version of the Big 5 and the remainder completed it online. Of the 219, eighteen were medical students and were removed from the analyses, leaving a final comparison sample of 201 (mean age = 19.7 years, SD = 1.5, 46% male; 32% were humanities, 9% engineering, 25% science and 34% social science students). The analyses reported here focus on the pencil and paper version as it is comparable, in terms of method of administration with the version used by the main sample. Furthermore, scores on ES and S differed significantly between the non-medical students who completed the paper and pencil versus the online versions (Ns = 201 and 237 respectively). The paper and pencil scores are higher on S (43.8 vs 41.0 respectively, *p* = .001) and ES (41.9 and 41.0 respectively: *p*<.001).

### Measures

#### Personality

The Big 5 personality traits were assessed using Goldberg's [27] 35 bi-polar markers of Surgency (S), Agreeableness (A), Conscientiousness (C), Emotional Stability (ES) and Intellect (I). Each scale is based on 7 items scored on a 9 point Likert-type bipolar adjective scales.

#### Prior Academic Knowledge

This was assessed in terms of results of two sets of National knowledge based exams sat at 16 (GCSE) and 18 (A levels) years of age. Students may take a large number of GCSEs covering sciences, art, social sciences and humanities but usually take 3 A levels. GCSE and A levels are tests of basic knowledge and are assessed by a mixture of course work and written exams. Students are offered places at Nottingham medical school a based on a specified minimum level of achievement at GCSE and then on their A level grades. GCSEs and A levels are converted to an alpha-numeric tariff system. For GCSE, an A grade equates to 12 points, a B to 10 and so on; and for A levels an A to 10 points, a B to 8 and so on.

#### Intelligence

This was assessed using the Personal Qualities Assessment (PQA) Mental Agility Test (MAT) [45]–[46]. The test has 45 MCQs with one point recorded for each correct answer. High scores indicate greater spatial, verbal and arithmetic ability.

#### Medical School Performance

Pre-clinical knowledge was assessed with a composite score over the first 2 years. This assesses knowledge about anatomy, physiology, biology, social science and diagnostics via Multiple Choice Questions (MCQs), written assessment and case reports. The year 3 assessment consists of a composite score based on exam performance and an empirical project. This represents the Bachelor of Medical Science (B Med Sci) and is an additional index of pre-clinical knowledge. The final two years represent clinical training. These distinguish clinical knowledge from clinical skills. Clinical knowledge is assessed by six composite knowledge based MCQs and short answers assessments. These six assessments are: Child Health, Specials (Ophthalmology, ENT, Dermatology), Health Care of the Elderly, Obstetrics and Gynaecology, Psychiatry and Advanced Clinical Experience. Clinical skills are assessed by OSCE/OSLER type composite skills assessments (Child Health, Obstetrics and Gynaecology, Psychiatry and Advanced Clinical Experience). All final composite marks are percentages.

### Ethical Approval

The main study was approved by the University of Nottingham Medical School Ethics Committee (F/11/2002). The comparison sample was taken from a study approved by University of Nottingham School of Psychology ethics committee (Date approved 09/05/08: VC/axg). All participants were over the age of 17 years and provided written informed consent to participate in the study as approved by the ethics committee. There were no minors or children involved in the study.

### Analyses

Initial descriptive analyses were completed using SPSS 20. Confirmatory factor analytic (CFA) and structural equation models (SEM) were conducted in M*Plus 7* [47].

#### Confirmatory Factor Analyses of Medical Skills and Knowledge

There were 12 assessments in total consisting of two pre-clinical knowledge assessment (knowledge score for years 1&2 and BMedSci), as well as six clinical knowledge assessments and four clinical skills assessments. We compared the fit of the four different models detailed in the introduction: (1) a single factor model, (2) a ‘two factor temporal model’ [13]), (3) a ‘two factor knowledge and skills model’ and (4) a ‘three factor model’.

#### Curvilinear Effects

Initially we explored for curvilinear effects of all the Big 5 traits on pre-clinical knowledge, clinical knowledge and clinical skills. Evidence for curvilinear effects was examined by running a series of hierarchical linear regressions, regressing pre-clinical knowledge (years 1 and 2 and B Med Sci separately), clinical knowledge (sum of the 6 assessments) and clinical skills (sum of the 4 assessments) on the linear and quadratic terms for each trait. The linear term was entered at step 1 and the quadratic term at step 2. A significant improvement in fit from step 1 to step 2 is taken as evidence of a curvilinear effect [48]. If there was evidence for curvilinear effects these were included in the SEMs.

#### SEM of Traits Predicting Medical Training

It has been argued that failure to model the underlying learning process is a major limitation of research in this area [13], [49]–[51]. Thus the models examined here have a ‘learning backbone’, as defined by McManus et al [13], to reflect how current learning is dependent of learning at an earlier stage. The learning backbone starts with the first exams the students took (GCSE at 16), followed by their A levels (at 18), then to the first 2 years of pre-clinical assessments, their year 3 B Med Sci assessments, and finally their clinical knowledge and clinical skills assessed in the final two years.

It has been argued that effects of personality, intelligence and demographics should be added to this backbone [13], [20]. We achieved this by a mixture of theoretical specified paths combined with more exploratory analyses. Theoretically, we specified (1) C to influence the whole learning process [6]–[7], with C predicted to have a negative effect on clinical knowledge and a positive effect on pre-clinical knowledge [20], (2) ES to effect clinical skills, with the prediction that this would be a negative association [9], (3) intelligence to influence the whole leaning backbone, with the prediction that higher levels of intelligence would enhance learning [12] and (4) sex and ethnicity to influence clinical skills and knowledge, with women and white students performing better [40]–[42]. For the exploratory component, paths from the remaining Big 5 traits and intelligence were specified to influence all components of the learning backbone from GCSEs to clinical skills and clinical knowledge (any identified quadratic terms were also added). The Big 5 was also specified to predict intelligence. This model was termed the ‘Full Model’. Non-significant paths between traits and intelligence and from traits and intelligence to the learning backbone were deleted using backward deletion until only significant paths remained. However, non-significant paths across the learning backbone were retained [13]. This was because we wanted to highlight the complete learning process in the final model (so the differential effects of distal learning on later learning can be observed) and how this is influenced specifically by personality and intelligence. Thus the resulting ‘Final Model’ had the complete learning backbone, and indicated how this was significantly influenced by intelligence and personality. The effects of ethnicity and sex were also included without deleting non-significant paths, again so as the relative effect sizes could be observed.

All models were estimated using maximum likelihood estimation with robust standard errors. Model fit was assessed in terms of the root mean square error of approximation (RMSEA) which should be less than .05 (the probability that the attained value of RMSEA was different to a RMSEA of .05 was also examined, and this should be non-significant), the square root mean residual (SRMR) which should be less than .06, and the Tucker-Lewis Index (TLI) and Comparative Fit Index (CFI) which should be .96 or greater [52]. The Akaike Information Criteria (AIC) was used to examine fit between models, with smaller values indicating better fit.

## Results

### Descriptives

The means, standard deviations, ranges and reliabilities for the personality variables for the main and comparison sample are presented in Table 2. The medical student sample scored significantly higher on 4 of the Big 5 domains compared to the comparison sample (S, A, C and ES, all *F*s>39.18 and all *p*s<.001), but there was no significant difference for I (*F*(1, 419) = 0.41, *p* = .52). Medical students were more extraverted (surgency), agreeable, conscientious and emotionally stable. Examining the ranges also indicates that in the medical student sample participants were less likely to endorse the lower end of the potential range of scores.

**Table 2:** Means, Standard Deviations (SDs), Ranges and Reliabilities for Personality Scores.

		Medical Students (N = 220)				Non- Medical Students (N = 201)			
		Mean	SD	Range	Cronbach's Alpha	Mean	SD	Range	Cronbach's Alpha
Intelligence									
	PQA Mental Agility	28.8	4.5						
Personality									
	Surgency (S)	47.1	6.2	26, 61	.82	43.8	8.6	17, 63	.83
	Agreeableness (A)	53.0	4.9	35, 63	.82	47.7	8.5	15, 63	.86
	Conscientiousness (C)	50.9	5.7	34, 62	.80	43.9	9.5	15, 63	.86
	Emotional Stability (ES)	46.6	6.5	24, 61	.78	41.9	9.0	15, 63	.81
	Intellect (I)	49.8	5.4	34, 63	.72	49.4	7.7	19, 63	.82

Note. The potential full range on each of the personality measure is 7 to 63.

The descriptive statistic for intelligence and the assessments are shown in Table 3.

**Table 3:** Means and Standard Deviations (SDs) for Exams.

Assessment		Mean	SD
Previous Academic Knowledge			
	GCSE	11.1	0.61
	A level	9.7	0.61
Pre-Clinical Knowledge			
	Years 1–2	64.5%	7.4
	BMedSci (year 3)	65.5%	4.5
Clinical Knowledge (Years 4–5)			
	Child Health	66.2%	6.1
	‘Specials’*	63.4%	10.0
	Health Care of the Elderly	61.5%	11.7
	Obstetrics and Gynaecology	64.6%	7.2
	Psychiatry	64.1%	7.6
	Advanced Clinical Examination	67.9%	4.3
Clinical Skill (Years 4–5)			
	Child Health	69.9%	4.3
	Obstetrics and Gynaecology	67.4%	8.5
	Psychiatry	57.5%	9.3
	Advanced Clinical Examination	69.4%	6.3

*Note*. N = 220, except GCSE based on 201 and A levels on 215.

‘Specials’ comprises Ophthalmology, Otolaryngology and Dermatology.

### Distinguishing Skill and Knowledge in the clinical course

The fit statistics for the 4 confirmatory factor models are given in Table 4. As can be seen the three factor model is the best fitting model (lowest AIC, highest CFI and TLI, lowest RMSEA and RMSR). As the 2 two-factor models and the ‘three factor model’ are nested the chi-square difference test was also calculated (adjusted for the use of the MLR estimator) to see if the ‘three-factor model’ represented an improvement in fit. This showed that the chi-square value for the ‘three factor model’ was significantly lower than both the two factor models (*ps*<.001). As such, the three factor model is a significant improvement in fit over the two factor models. The three factor model is shown in Table 5.

**Table 4:** Fit Statistics for the CFA models.

	χ^2^	CFI	TLI	RMSEA	RMSR	AIC
Model						
One factor	153.74 (54), *p* = .96	.89	.87	.092*	.064	17403.667
Two Factors - Temporal	116.8 (53), *p* = .95	.93	.92	.07*	.058	17368.96
Two Factors – Knowledge and Skill	98.02 (53), *p* = .96			.062	.045	17352.31
Three Factors – Pre-clinical knowledge. Clinical knowledge and clinical skill	62.02 (51), *p* = .95	.99	.98	.031	.037	17320.61

*Note*.

*  = Indicates if the probability that RMSEA is ≤.05.

**Table 5:** Confirmatory Factor Model for Clinical Skills and Knowledge.

	Years in which assessed		
	Years 1–3	Years 4–5	
	Pre-Clinical Knowledge	Clinical Knowledge	Clinical Skills
Assessment			
Knowledge Years 1–2	.77***		
BMedSci	.83***		
Child health - knowledge		.75***	
Specials (ophthalmics, ENT, dermatology) - Knowledge		.74***	
Health care of the elderly - knowledge		.53***	
Obstetrics and Gynaecology - knowledge		.62***	
Psychiatry - knowledge		.79***	
Advanced Clinical Examination - knowledge		.82***	
Child health - skills			.41***
Obstetrics and Gynaecology - skills			.43***
Psychiatry - skills			.67***
Advanced Clinical Examination - skills			.69***

*Note*.

*** *p*<.001. *N* = 220.

### Personality, Intelligence and Demographics Predicting Learning Across the Medical Context

#### Curvilinear effects

**Figure 1:**
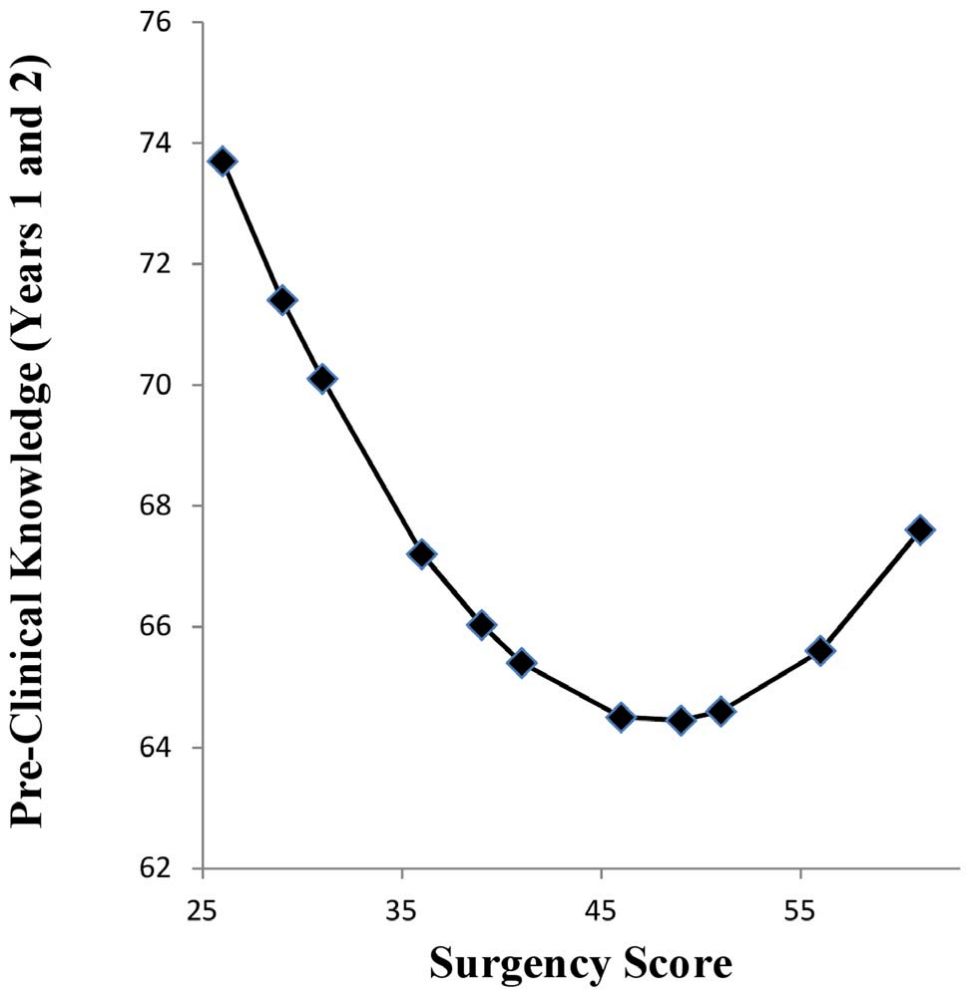
Curvilinear Effect of Surgency of Pre-Clinical Knowledge Acquisition on Years 1 and 2.

There was evidence for a curvilinear effect of S on pre-clinical knowledge assessed in years 1 and 2. The linear effect at step 1 was non-significant (*R^2^* = .013, *B* = −1.83, *p* = .095), however, there was a significant improvement in fit with the addition of the quadratic term for S at step 2 (Δ*R* ^2^ = .02, *p* = .039, *B* = 0.019, *p* = .039). This curvilinear effect is shown in Figure 1. This is a U shaped function, with performance best at lower levels S (intraversion), decreasing through mid-range levels of S and improving again as S starts to increase. This curvilinear effect of S was included in the SEMs with respect to predicting pre-clinical knowledge in years 1 and 2. The linear term for S with respect to pre-clinical knowledge in years 1 and 2 was not included as it is non-significant. There were no other curvilinear effects for any other Big 5 traits on any of the learning outcomes.

#### Main Model Fitting

**Figure 2:**
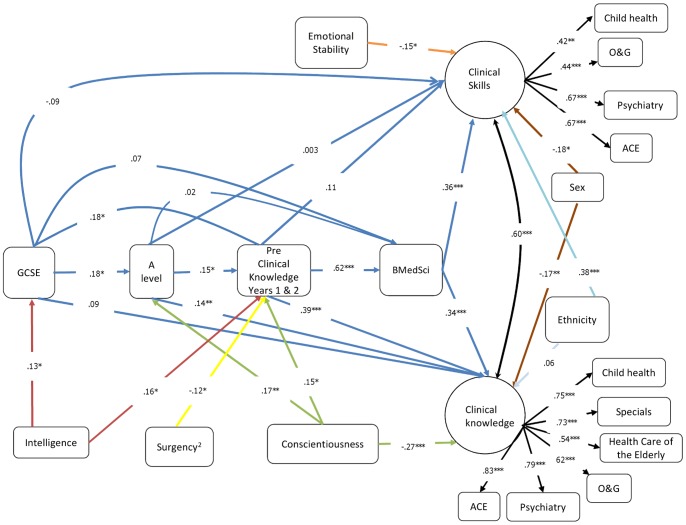
Structural Equation Model of Personality and Intelligence on Knowledge and Skill Acquisition in Medical Training. Ethnicity (0 = non-white and 1 = white), Sex (0 = female and 1 = male). Dark blue lines represent the ‘learning backbone’, green lines represent the effects of conscientiousness, the yellow line represents the effect of surgency^2^, red lines represent the effects of intelligence, the orange line represents the effect of emotional stability, light blue lines represent the effects of ethnicity, brown lines represent the effects of sex, black lines represent factor loadings and the correlation between latent factors. O&G = Obstetrics and Gynaecology, Special (Ophthalmology, Otolaryngology and Dermatology), ACE = Advanced Clinical Examination. Coefficients are standardized. * p<.05, ** p<.01, *** p<.001.

The full correlation matrix for these analyses can be found in Table S1 in the supplementary files. The ‘Full Model’ was a good fit to these data (CFI = .966, TLI = .953, RMSEA = .032 (probability that RMSEA≤.5 is .984), SRMR = .044; χ^2^ = 198.26 (162), *p* = .027; AIC = 19592.226). Consistent with the hypothesized effects, C had a positive effect on pre-clinical knowledge (β = .16, *p* = .025) and negative effect on clinical knowledge (β = −.26, *p*<0001), but was not significantly associated with clinical skills (β = .07, *p* = .35). Consistent with the defensive direction account, ES had a negative effect on clinical skill (β = −.21, *p* = .008), but no significant effect on clinical knowledge (β = .03, *p* = .68). The non-significant paths for personality and intelligence were deleted from this model. However, we retained two marginally significant effects, one for A on GCSE scores (β = .16, *p* = .055) and the quadratic effect of S on preclinical knowledge in years I and 2 (β = −.12, *p* = .068). As all other non-significant effects had p-values greater than .153, we decided to retain these two effects to ensure we were not missing any important effects (see [53]). In the resulting subsequent model the effect of A remained non-significant (β = .01, *p* = .095) but the quadratic effect of S became significant (β = −.12, *p* = .034). Thus A was deleted but the quadratic effect of S retained. The resulting ‘Final Model’ (Figure 2) was a good fit (CFI = .966, TLI = .959, RMSEA = .034 (probability that RMSEA≤.5 is .964), SRMR = .046; χ^2^ = 180.79 (144), *p* = .02; AIC = 18258.28). The AIC suggests the ‘Final Model’ is a better fit than the ‘Full Model’. The medical school exam board also have a differential weighting scheme for the knowledge and skills exams. When these data are analysed such that manifest weighted clinical knowledge and skills variables are used the same general pattern of results emerges.

Examining the ‘learning backbone’ (blue paths in Figure 2) in more detail shows, consistent with McManus et al [13], that subsequent learning in medical school is predicted by the adjacent preceding learning, with the pre-clinical knowledge also having a carry forward effect predicting clinical knowledge. Importantly, and again consistent with McManus et al [13], general knowledge (A levels & GCSEs) also carry forward in that they not only predict their most adjacent learning outcome (GCSEs predict A levels, and A levels predict pre-clinical knowledge) but also more distal learning at medical school: GCSEs predict ‘pre-clinical knowledge’ and A levels predict ‘clinical knowledge’.

Interestingly clinical knowledge was predicted by two distal knowledge assessments (A levels and pre-clinical knowledge), whereas clinical skills were not predicted by these. Indeed, the effect of A levels on clinical knowledge is significantly different from its effect on clinical skills (*p* = .019) and similarly the effect of pre-clinical knowledge on clinical knowledge and was significantly greater than its effects on clinical skills (*p*<.001). This indicates that while knowledge based outcomes are good predictors of each other, clinical skill is not predicted by knowledge (general or clinical). To examine this further the total and total indirect effects of A levels on clinical knowledge and clinical skills were calculated. For clinical knowledge both the total (β = .24, *p*<.001) and total indirect effects (β = .10, *p* = .037) were significant and positive. However, for clinical skills both the total (β = .06, *p* = .49) and total indirect effects (β = .06, *p* = .07) were non- significant. This indicates that clinical knowledge but not clinical skill are predicted from A levels.

Confirming the cost-benefit hypothesis for C, C was a positive predictor of pre-clinical knowledge (and A levels) and a negative predictor of clinical knowledge. To further explore the effect of C, its total and total indirect effects on clinical skill and clinical knowledge were calculated. The total indirect effect of C on clinical knowledge was significant and positive (β = .13, *p* = .003), and total effect of C on clinical knowledge was significant and negative (β = −.14, *p* = .025). Thus, while indirectly C has a positive effect on clinical knowledge, overall the effect of C on clinical knowledge is negative. In support of the defensive direction hypotheses for moderate-low ES, ES was negatively associated with clinical skills. The curvilinear effect of S on pre-clinical knowledge was also significant.

The effects of intelligence are limited to early learning, with intelligence predicting GCSE and pre-clinical knowledge but unrelated to the rest of the learning process. However, interestingly, intelligence has a significant and positive total indirect effect on both clinical knowledge (β = .13, *p* = .002) and clinical skills (β = .055, *p* = .03).

The standard effects of sex are observed, with women performing better than men on both clinical skills and clinical knowledge (the difference in effect size between clinical skills and knowledge for sex is non-significant: *p* = .86). Similarly effects of ethnicity were observed with white students performing better at clinical skills than non-white students (the size of the effect of ethnicity on clinical skills was significantly greater than on clinical knowledge: *p*<.0001).

## Discussion

The results of this study show that, consistent with the cost-benefit account of personality [11], conscientiousness (C) enhances performance when the context requires methodical and ordered thought, but reduces it when the context requires flexibility of thought. The study also provides evidence for a ‘bright side’ to moderate anxiety [9], and demonstrates that pre-clinical knowledge, clinical knowledge and clinical skill are separable psychometrically and differentially predicted by personality and previous knowledge [15].

### The ‘Dark Side’ of Conscientiousness and the ‘Bright Side’ of Anxiety

Applied personality research has long suggested that C carries benefits when selecting into organizations [6]–[7]. A problem with this validity generalizability approach, as highlighted by these results, concerns the changing face of the work environment. The nature and structure of jobs will change as an employee progresses through their career or as technology changes. Thus, simply suggesting that a trait may be generally beneficial requires some reconsidering, and highlights the need to consider the ‘dark side’ of traits like C [8], [20]. This is important as it has been mooted that C is considered a key trait when selecting medical students [33]. The results reported here suggest that this may require further consideration [54]. It should also be noted that our sample of medical students tended to score towards the higher end of the measure of C used in this study. As such, they are more likely to reflect both normal-high and maladaptively high levels of C [28].

The results also provide some evidence for the defensive direction account of anxiety [9]. Clinical skill acquisition and assessment are rated as stressful [24] and greater anxiety was associated with greater clinical skill acquisition. The results show clearly that even once prior learning is controlled, those who have moderately higher levels of anxiety (low ES) perform better on the more anxiety-provoking part of the course. Again considering the range of scores on ES in our medical student sample, they tended not to score at the very low end, which reflects high neuroticism/anxiety, but rather reflect moderate anxiety. Thus, our sample may include students with normal high levels of neuroticism/anxiety as suggested by Widiger and Mullins-Sweatt [28], reflecting greater vigilance and supporting the tendency to move towards the object of anxiety to control it [9].Thus, there is a ‘bright side’ to anxiety, at these moderate levels.

We also observed a U shaped function linking S to pre-clinical knowledge in years 1 and 2. This function suggests that low levels of S are associated with better performance, which reduces as S increase, and then increases again for higher levels of S. This association was not predicted and thus requires replication. However, we can offer some speculative interpretation of this U shaped function. In terms of the range, S in our sample did not stretch to the extreme low end, but again was moderately low. Based on Widiger and Mullins-Sweatt [28] we can tentatively suggest that that this may reflect a cautious, serious and more formal approach to life and these may be attributes that are helpful in a learning context that is more formal and ordered. Indeed, we observed that for pre-clinical knowledge (in years 1 and 2), not only is it moderately lower levels of S that enhance performance here, but also higher levels of C and intelligence. These pre-clinical years seem to be a focal point representing the confluence of effects of S, C and intelligence on subsequent learning. Furthermore, based on Eysenckian [55] arousal theory, the intravert is more likely to seek out quiet and calm situations (e.g., libraries) and these types of context should also enhance opportunities to learn. The slight improvement in performance at higher levels of S may reflect drive and energy linked to higher levels of S.

Intelligence was shown to have its major effect early in the learning process [56] and this is consistent with previous reports by McManus et al [39]. However, the results also show, that intelligence had a positive total indirect effect on both clinical knowledge and clinical skills. A levels also had positive direct and total indirect effects on clinical knowledge but no effect on clinical skills. These findings have important implications for those who argue that selection into higher education should be on the basis of intelligence rather than knowledge assessment [57]. These findings suggest that both are important and need to be considered together rather than as alternatives.

### Skill and Knowledge

We show that the acquisition of pre-clinical knowledge, clinical skills and clinical knowledge are, not only psychometrically separable, but also have different predictors. Clinical knowledge is predicted by prior academic knowledge and negatively by C. Clinical skills, on the other hand, are predicted by the most proximal learning outcome, B Med Sci and moderately higher levels of anxiety [9]. The important applied point here is that, when considering medical selection, differential predictors of skill and knowledge should be built into selection models. Clinical knowledge and clinical skills are still developing and will become more ‘tuned’ [14] as their medical training continues into post-graduate specialization, but should still be psychometrically separate [19]. What would be interesting to learn is how knowledge is organized and structured as doctors become more ‘expert’ and their knowledge is tuned [19]. With increased expertise they should be able to make more useful links in ways they were not able to at earlier stages of their training. Techniques like multi-dimensional scaling and cognitive-structural mapping could be used to explore how medical knowledge is reorganized with increasing expertise [58]–[59].

### Conclusion

This paper shows that the association between one personality trait (i.e., conscientiousness) and learning outcomes may change in direction (from enhancing to inhibiting) as context changes. From an applied perspective this indicates that simply selecting on a trait – on the assumption that it will always confer benefits – needs re-evaluating, as a trait like conscientiousness may have a ‘dark side’. Conversely this study also shows that traits often believed to have a negative effect on outcomes (e.g., anxiety) can also have a ‘bright side’: positively predicting skill acquisition. The results highlight the need to be sensitive to the range of scores on the index of personality.

The paper also shows that clinical knowledge and skill are separable and predicted by different patterns of prior learning and personality. Again from an applied perspective, this implies that selection models need to consider the different type of learning outcome when being developed.

## Supporting Information

Table S1 **Estimated zero-order correlations between study variables.** *Note*. N = 220 based on Full Information Maximum Likelihood (FIML) estimation to account for missing data. This is the matrix that is the basis of the CFA and SEM models ran in the paper. Correlations greater than .14 are significant at p<. 05. PCK = Pre-clinical knowledge in years 1 and 2, BMedSci = Bachelor of Medical Science, know = Knowledge, skill = Skill, CH = Child Health, Specials comprises Ophthalmology, Otolaryngology and Dermatology, HCE = Health Care of the Elderly, OG = Obstetrics and Gynaecology, PSY = Psychiatry, ACE = Advanced Clinical Examination, Sex (0 = female and 1 = male), Ethnicity (0 = non-white, 1 = white); C = conscientiousness, ES = emotional stability, S = Surgency, A = agreeableness, I = Intellect, S^2^ = Surgency^2^.
(DOCX)
