# Supplementary material for: The ‘Dark Side’ and ‘Bright Side’ of Personality: When Too Much Conscientiousness and Too Little Anxiety Are Detrimental with Respect to the Acquisition of Medical Knowledge and Skill
Source: PLoS One. 2014 Feb 27;9(2):e88606. doi: 10.1371/journal.pone.0088606 (PMC3937323; doi:10.1371/journal.pone.0088606)
Supplement: Table S1 — Estimated zero-order correlations between study variables. Note. N = 220 based on Full Information Maximum Likelihood (FIML) estimation to account for missing data. This is the matrix that is the basis of the CFA and SEM models ran in the paper. Correlations greater than .14 are significant at p<. 05. PCK = Pre-clinical knowledge in years 1 and 2, BMedSci = Bachelor of Medical Science, know = Knowledge, skill = Skill, CH = Child Health, Specials comprises Ophthalmology, Otolaryngology and Dermatology, HCE = Health Care of the Elderly, OG = Obstetrics and Gynaecology, PSY = Psychiatry, ACE = Advanced Clinical Examination, Sex (0 = female and 1 = male), Ethnicity (0 = non-white, 1 = white); C = conscientiousness, ES = emotional stability, S = Surgency, A = agreeableness, I = Intellect, S2 = Surgency2. (DOCX) [file pone.0088606.s001.docx]

Table S1. Estimated zero-order correlations between study variables

|  | 1 | 2 | 3 | 4 | 5 | 6 | 7 | 8 | 9 | 10 | 11 | 12 | 13 | 14 | 15 | 16 | 17 | 18 | 19 | 20 | 21 | 22 |
| --- | --- | --- | --- | --- | --- | --- | --- | --- | --- | --- | --- | --- | --- | --- | --- | --- | --- | --- | --- | --- | --- | --- |
| GCSE (1) | 1 |  |  |  |  |  |  |  |  |  |  |  |  |  |  |  |  |  |  |  |  |  |
| A level (2) | .18 | 1 |  |  |  |  |  |  |  |  |  |  |  |  |  |  |  |  |  |  |  |  |
| Intelligence (3) | .13 | .02 | 1 |  |  |  |  |  |  |  |  |  |  |  |  |  |  |  |  |  |  |  |
| PCK (4) | .22 | .21 | .19 | 1 |  |  |  |  |  |  |  |  |  |  |  |  |  |  |  |  |  |  |
| BMedSci (5) | .19 | .16 | .13 | .64 | 1 |  |  |  |  |  |  |  |  |  |  |  |  |  |  |  |  |  |
| CH-Know (6) | .22 | .18 | .10 | .41 | .48 | 1 |  |  |  |  |  |  |  |  |  |  |  |  |  |  |  |  |
| CH-Skill (7) | -.06 | -.06 | .04 | .12 | .22 | .10 | 1 |  |  |  |  |  |  |  |  |  |  |  |  |  |  |  |
| Specials (8) | .17 | .17 | .10 | .45 | .52 | .58 | .09 | 1 |  |  |  |  |  |  |  |  |  |  |  |  |  |  |
| HCE-Know (9) | .21 | .02 | .14 | .30 | .26 | .38 | .12 | .37 | 1 |  |  |  |  |  |  |  |  |  |  |  |  |  |
| OG-Know (10) | .15 | .10 | .13 | .35 | .41 | .52 | .13 | .44 | .39 | 1 |  |  |  |  |  |  |  |  |  |  |  |  |
| OG-Skill (11) | .09 | -.006 | .07 | .21 | .24 | .24 | .19 | .16 | .20 | .19 | 1 |  |  |  |  |  |  |  |  |  |  |  |
| PSY-Know (12) | .26 | .21 | .11 | .49 | .51 | .59 | .20 | .56 | .46 | .47 | .19 | 1 |  |  |  |  |  |  |  |  |  |  |
| PSY-Skill (13) | .03 | .05 | .06 | .15 | .27 | .32 | .30 | .29 | .26 | .38 | .25 | .39 | 1 |  |  |  |  |  |  |  |  |  |
| ACE-Skill (14) | -.004 | .03 | .07 | .18 | .30 | .32 | .31 | .27 | .19 | .34 | .32 | .31 | .44 | 1 |  |  |  |  |  |  |  |  |
| ACE-Know (15) | .22 | .22 | .17 | .53 | .52 | .61 | .11 | .63 | .45 | .48 | .21 | .66 | .36 | .35 | 1 |  |  |  |  |  |  |  |
| Ethnicity(16) | -.02 | -.04 | .10 | -.08 | .08 | .04 | .15 | .04 | .12 | .19 | .23 | -.05 | .25 | .27 | .10 | 1 |  |  |  |  |  |  |
| Sex (17) | -.14 | .11 | .04 | .06 | -.07 | .18 | -.05 | -.06 | -.12 | -.16 | -.14 | -.17 | -.18 | -.07 | .02 | .08 | 1 |  |  |  |  |  |
| C (18) | .001 | .17 | -.09 | .14 | .03 | -.11 | .12 | -.08 | -.08 | -.06 | .02 | -.06 | .06 | -.05 | -.19 | -.10 | -.14 | 1 |  |  |  |  |
| ES (19) | -.06 | .03 | -.11 | .02 | -.03 | -.13 | -.03 | -.04 | -.05 | -.09 | -.08 | -.04 | -.09 | -.17 | -.10 | -.03 | .21 | .34 | 1 |  |  |  |
| S (20) | .04 | .03 | -.14 | -.11 | -.05 | -.09 | .01 | -.08 | -.20 | .02 | .03 | -.09 | .06 | .05 | -.15 | .04 | -.03 | .23 | .30 | 1 |  |  |
| A (21) | .09 | .07 | -.16 | .03 | .02 | -.03 | .02 | .05 | -.07 | -.04 | -.02 | .06 | -.004 | -.07 | -.06 | -.21 | -.13 | .40 | .37 | .29 | 1 |  |
| I (22) | -.03 | .01 | -.15 | -.06 | -.03 | -.13 | -.005 | -.03 | -.10 | -.10 | -.04 | -.11 | -.01 | -.10 | -.17 | -.12 | .07 | 38 | .37 | .37 | .35 | 1 |
| S² (23) | .04 | .04 | -.14 | -.10 | -.05 | -.09 | .01 | -.08 | -.20 | .03 | .03 | -.09 | .06 | .06 | -.15 | .04 | -.03 | .25 | .30 | .99 | .29 | .38 |

*Note*. N = 220 based on Full Information Maximum Likelihood (FIML) estimation to account for missing data. This is the matrix that is the basis of the CFA and SEM models ran in the paper. Correlations greater than .14 are significant at p <. 05. PCK = Pre-clinical knowledge in years 1 and 2, BMedSci = Bachelor of Medical Science, know = knowledge, skill = skill, CH = Child health, Specials comprises Ophthalmology, Otolaryngology and Dermatology, HCE = Health care of the elderly, OG = Obstetrics and Gynaecology, PSY = Psychiatry, ACE = Advanced Clinical Examination, Sex (0 = female and 1 = male), Ethnicity ( 0 = non-white, 1 = white); C = conscientiousness, ES = emotional stability, S = Surgency, A = agreeableness, I = Intellect, S² = Surgency²
